# Does Anxiety Increase the Risk of all-Cause Dementia? An Updated Meta-Analysis of Prospective Cohort Studies

**DOI:** 10.3390/jcm9061791

**Published:** 2020-06-09

**Authors:** Javier Santabárbara, Darren M. Lipnicki, Beatriz Olaya, Beatriz Villagrasa, Juan Bueno-Notivol, Lucia Nuez, Raúl López-Antón, Patricia Gracia-García

**Affiliations:** 1 Department of Preventive Medicine and Public Health, Universidad de Zaragoza, 50009 Zaragoza, Spain; jsantabarbara@unizar.es (J.S.); 738473@unizar.es (L.N.); 2 Instituto de Investigación Sanitaria de Aragón (IIS Aragón), 50009 Zaragoza, Spain; rlanton@unizar.es; 3 Centro de Investigación Biomédica en Red de Salud Mental (CIBERSAM), Ministry of Science and Innovation, 28029 Madrid, Spain; 4 Centre for Healthy Brain Ageing, School of Psychiatry, University of New South Wales Medicine, Randwick, NSW 2052, Australia; d.lipnicki@unsw.edu.au; 5 Research, Innovation and Teaching Unit, Parc Sanitari Sant Joan de Déu, Universitat de Barcelona, 08830 Sant Boi de Llobregat, Spain; 6 Psychogeriatry, CASM Benito Menni, 08830 Sant Boi de Llobregat, Spain; beavibla@gmail.com; 7 Psychiatry Service, Hospital Universitario Miguel Servet, 50009 Zaragoza, Spain; elecrijuan@hotmail.com (J.B.-N.); pgraciagarcia@yahoo.es (P.G.-G.); 8 Department of Psychology and Sociology, Universidad de Zaragoza, 50009 Zaragoza, Spain

**Keywords:** dementia, anxiety disorder, risk factor, cohort study, meta-analysis

## Abstract

**Background:** Anxiety has been suggested as a potentially modifiable risk factor for dementia, but results are still controversial. Our main objectives are to develop an updated meta-analysis of prospective population-based studies on the relationship between anxiety and risk of dementia, and to estimate the population fraction of dementia attributable to anxiety (PAF). **Methods:** We searched for cohort studies listed on PubMed or Web of Science from January 2018 to January 2020 that reported risk estimates for the association between anxiety and incident dementia. These were added to cohort studies published before January 2018 that were used in a previously published meta-analysis. Fully adjusted RRs were pooled using random effects models. We estimated the proportion of incident dementia attributable to anxiety by using PAF. **Results:** The meta-analysis included nine prospective cohorts from eight studies, representing 29,608 participants. The overall relative risk (RR) of dementia was 1.24 (95% CI: 1.06–1.46) and the PAF of dementia due to anxiety was 3.9%. **Conclusions:** Anxiety is significantly associated with an increased risk of all-cause dementia. The treatment or prevention of anxiety might help to reduce dementia incidence rates, but more research is needed to clarify whether anxiety is a cause of dementia rather than a prodrome.

## 1. Introduction

Dementia has been considered a public health priority by the World Health Organization (WHO), due to the growing number of subjects suffering the disease around the world and its burden for patients, their families and society [1]. Due to the irreversible nature of the disease and the lack of effective treatment [2], identifying potentially modifiable risk factors for dementia to design preventive strategies has become a research priority [3,4]. Thus, despite the upward trend in the estimates of dementia prevalence [5], in recent decades, several epidemiological studies conducted in high-income countries [6,7,8,9] suggest stabilization and even a decline in the prevalence and incidence of dementia. This stabilization might be explained by preventive strategies targeting modifiable risk factors for dementia [2].

A recent systematic review shows that around 35% of dementia is attributable to the combination of several modifiable risk factors, including cardiovascular risk factors, depression or educational attainment [10]. Anxiety has also been recognized as one of the potentially modifiable dementia risk factors [11]. However, the results in the literature are controversial, with a recent systematic review suggesting that anxiety might be a risk factor for dementia [12], but other studies did not find this association to be significant [13]. The variability of results between studies might be partly explained by methodological differences in the anxiety measure, duration of the follow-up period, and the lack of adjustment for potentially significant covariates in the association between anxiety and dementia, such as depression. Another possible reason for the variability in the results might be found in the diversity of populations. A meta-analysis of six studies by Gulpers et al. [14] reported that older adults with anxiety had a 57% higher risk of developing dementia. The risk is even higher for anxiety with a late-life onset, which might indicate that anxiety in older adults would be a prodromal sign of dementia. However, this meta-analysis included both cognitively healthy samples as well as samples with mild cognitive impairment. In a previous meta-analysis exclusively using population-based cohort studies with cognitively intact participants [15], we found that anxiety conveyed a 29% increased risk of dementia. 

Since our meta-analysis in 2018, further papers on the link between anxiety and risk of all-cause dementia have been published [16,17,18]. The aims of the present study are to provide an up-to-date estimation of the association between anxiety and risk of all-cause dementia and calculate the population-attributable fraction (PAF) of dementia due to anxiety.

## 2. Methods

This study was conducted in accordance with the PRISMA guidelines for reporting systematic reviews and meta-analyses [19].

### 2.1. Search Strategy and Selection Criteria

In October 2019, we conducted a search of all cohort studies reporting the association between anxiety and risk of all-cause dementia published at MEDLINE via PubMed and Web of Science (the search was repeated in January 2020 with the same results). Briefly, the search strategy included the following terms: (anxiety AND Dementia AND (cohort study OR longitudinal study OR incidence)) using both medical subject headings and free text (Supplementary Table S1). We only considered studies written in English and published after January 2018 to avoid overlap with our previous meta-analysis [15]. We also focused on prospective population-based studies with baseline assessment of anxiety in cognitively intact subjects who were re-evaluated at follow-up for incident all-cause dementia. 

### 2.2. Data Extraction and Quality Assessment

We extracted the following information from each study included in the meta-analysis (MA): country, sample size, number of prevalent cases of anxiety, the number of incident cases of all-cause dementia, percentage of women, mean age of the sample, instrument used to measure anxiety, tool and clinical criteria used to diagnose dementia, covariates included in the adjusted models, statistical model, adjusted RR estimates, and time of follow-up.

We used the Newcastle-Ottawa scale (NOS) for cohort studies [20] to analyse the quality of each study. This is a nine-point scale which evaluates the risk of bias of a given cohort study based on three criteria: population selection, comparability and outcome. Scores of 0–3, 4–6, and 7–9 indicate low, moderate, and high quality, respectively.

### 2.3. Data Analysis

Relative risk (RR) was used as measure of association. Risk estimates from fully adjusted models were preferentially pooled in our analyses. Cohen’s *d* was performed to illustrate the effect size of the differences in the risk of all-cause dementia between anxiety and non-anxiety groups. The effect size for overall RR and its confidence interval were calculated according to Sánchez-Meca et al. [21], and was classified as “small” (˜0.2), “medium” (˜0.5) or “large” (˜0.8) [22]. 

The Hedges *Q* statistic was used as a measure of heterogeneity (statistical significance was set at *p* < 0.10) and quantified with the *I^2^* statistic (high heterogeneity was considered as ≥75%) [23]. We performed random-effect model meta-analyses. Additionally, univariate meta-regressions were calculated to help identify potential sources of heterogeneity when estimating pooled RR [24], taking into account the following variables: mean age and percentage of women at baseline, sample size, duration of the follow-up period, and methodological quality. We also conducted a sensitivity analysis to inspect the influence of a single study on the overall result by omitting them one by one. The fail-safe *N* value was used as indicator of publication of bias [25]. This statistics is recommended when there are less than 10 studies in the MA [26,27] and indicates the number of non-significant, unpublished (or missing) studies that would be need to be added in the MA to reduce an overall statistically significant result to non-significance. Relatively large values of the fail-safe number, compared with the number of observed studies, indicate confidence in the summary conclusions [25]. In the event of the identification of publication bias in the pooled estimate, the overall RR would be adjusted using the ‘trim and fill’ method [28].

Finally, we estimated the proportion of incident all-cause dementia attributable to anxiety by calculating the PAF [29] and its confidence intervals were computed using the substitution method [30]. The PAF indicates the proportion of all-cause dementia cases that would be avoided if anxiety could be prevented, assuming a causal effect and unbiased estimates. The pooled prevalence of anxiety was calculated by combining the prevalence rates of selected studies in a random-effects meta-analysis model, as reported in previous work [31]. 

All the statistical analyses were performed with STATA statistical software (version 10.0; College Station, TX, USA) and R (R Core Team, 2019). 

## 3. Results

### 3.1. Study Selection

**Figure 1:**
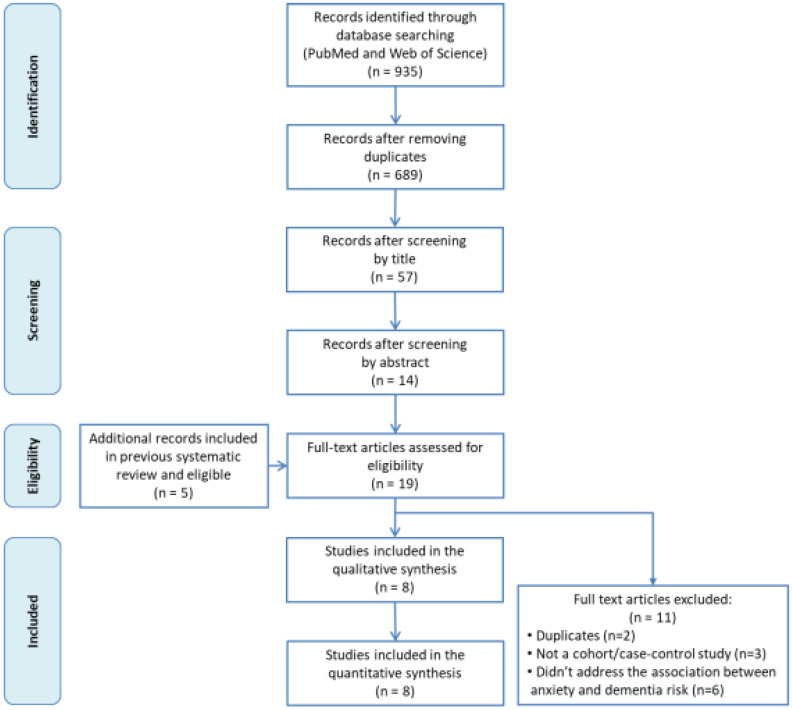
Flow chart of the literature search strategy and study selection process.

The flowchart of the search strategy and study selection process is shown in Figure 1. A total of 935 potential records were initially drawn from the search, from which 246 were duplicated and thus removed. Then, we read the titles and abstract of the remaining 689 articles. Some 675 did not meet the inclusion criteria and were excluded. After reading the full text of 14 articles, only three were finally selected. Additionally, the five studies from our previous systematic review were included [15].

### 3.2. Description of Included Studies

The eight included studies were published between 2009 and 2018, with nine prospective cohorts (two from de Bruijn et al. [13]) and a total of 29,608 participants. Table 1 displays the main characteristics of these studies. Five were from Europe [13,16,18,32,33], two from the United States [17,34] and one from Mexico [35]. One study included only women [34], and another included only men [32]. One study did not report the average age [32], but for the others, age ranged from 60.8 [33] to 82.8 years [34]. The duration of follow-up varied between 3 [35] and 28 years [33].

The studies used different scales to classify anxiety. Three used the State-Trait Anxiety Inventory (STAI) [16,32,33], and two assessed anxiety as a personality trait [16,32]. Anxiety symptoms were assessed with the Neuropsychiatric Inventory Questionnaire (NPI-Q) [35], the Hospital Anxiety and Depression Scale (HADS) [13], the Geriatric Anxiety Scale (GAS) [34] and the Beck Anxiety Inventory [17]. The Diagnostic and Statistical Manual of Mental Disorders (DSM-IV) [36] criteria (sample II of de Bruijn et al. [13]), and the Geriatric Mental State-Automated Geriatric Examination for Computer Assisted Taxonomy (GMS-AGECAT) [18] were used instead of an anxiety scale. 

The criteria used to diagnose dementia were more uniform across the studies, being based on the DSM in all but one, which used the modified Telephone Interview for Cognitive Status (TICSm) [17].

The studies also differed in the types of covariates that were included in the adjusted models. We calculated the pooled RR taking into account the RR estimated from the full adjusted models. The adjusted RR values ranged from 0.81 (95% CI: 0.50–1.30) [13] to 2.74 (95% CI: 1.18–6.35) [18].

### 3.3. Risk of Bias Assessment

Four studies presented low risk of bias (7–9 from a maximum of nine points) [13,16,18,35] and three had a medium risk of bias [17,32,34] (Table 1, Supplementary Table S2).

### 3.4. Meta-Analysis of Incidence Rates of all-Cause Dementia

**Figure 2:**
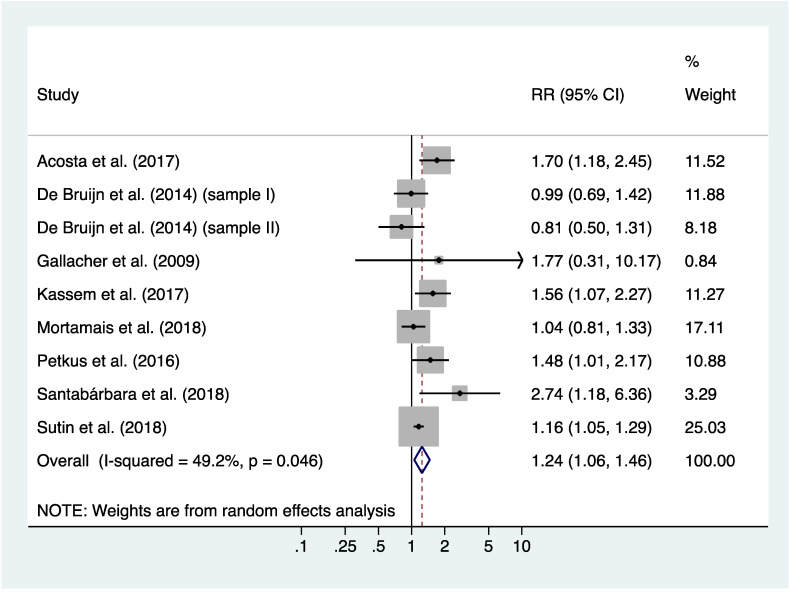
Meta-analysis of risk ratios for the association between anxiety and all-cause dementia.

Figure 2 shows the pooled results for the nine included cohorts of cognitively intact participants at baseline. Participants with prevalent anxiety at baseline had a 24% higher adjusted risk of incident dementia than those without anxiety (pooled RR: 1.24, 95% CI: 1.06–1.46; *p*=0.009). However, the effect size was small, with a Cohen’s *d* equal to 0.12 (95% CI: 0.03–0.21).

The results of individual studies were moderately heterogeneous (*Q*=15.76, df = 8, *p* = 0.046; *I^2^*=49.2%, 95% CI: 0–76%) (Figure 2). However, after excluding studies one-by-one from the analysis, the pooled RR slightly changed but remained statistically significant, from 1.16 (95% CI: 1.07–1.27) to 1.24 (95% CI: 1.08–1.42) (Supplementary Figure S1), indicating that none of the included studies had a disproportionate impact on the pooled RR.

### 3.5. Meta-Regression

After conducting meta-regression analyses, we found no significant association between the outcome and age, gender, sample size, follow-up duration, or methodological quality (see Table 2). This indicates that none of these covariates had a potential impact on the pooled RR.

### 3.6. Risk of Publication bias

The fail-safe *N* was 30, indicating that 30 studies with a null result would be needed to reduce the overall RR to a non-significant value. This would indicate absence of publication bias. Furthermore, the ‘trim and fill’ method to adjust for publication bias had a marginal effect on the pooled RR (RR: 1.21; 95% CI: 1.02–1.42) (Supplementary Figure S2), which remained statistically significant (*p* = 0.029).

### 3.7. Population Attributable Fraction

Prevalence rates of anxiety were pooled across eight of the nine selected cohorts (no data for Sutin et al. [17] were available) and then used in a random effects model. The estimated overall prevalence of anxiety was 20% (95% CI: 10%–31%), yielding a PAF of dementia due to anxiety of 3.9% (95% CI: 1.9%–6.0%).

## 4. Discussion

### 4.1. Main Findings

The present meta-analysis conducted with nine cohorts indicates a positive association between anxiety and risk of all-cause dementia. Participants with prevalent anxiety at baseline show 24% higher risk of developing dementia during the follow-up. Despite this being considered a low effect size, with an average of 20% of people with anxiety, the proportion of incident dementia attributable to anxiety is estimated to be 4%. This is also comparable to the proportion attributable to other modifiable risk factors such as diabetes or hypertension, which show a PAF of 3.2% and 5.1%, respectively [10].

### 4.2. Comparison with Previous Studies

This current work extends the findings of our previous meta-analysis [15] by including three new cohort studies [16,17,18] that yielded a sample of 29,608 older adults without dementia at baseline. The association between anxiety and an increased risk of all-cause dementia reported here is consistent with our previous meta-analysis, and with other studies [12,14,15]. Our current findings also complement the study by Gulpers et al. by providing the PAF of dementia due to anxiety.

The risk estimation found by Gulpers et al. [14] was twice as high as what we found in this study and our previous meta-analysis [15]. The main reason for this difference could be that three out of six community samples investigated by Gulpers et al. were comprised entirely of individuals with mild cognitive impairment at baseline, and who might have a higher risk of conversion to dementia than cognitively intact subjects [37]. In our meta-analysis, all studies excluded prevalent demented participants, and all but one [13] excluded participants with cognitive impairment at baseline (e.g., measured with the MMSE) or controlled for baseline cognition in the analysis.

Our results are consistent with a recent systematic review [12] that found a positive association between clinically significant anxiety and future dementia. This review included high-quality studies with a mean follow-up of at least 10 years to minimize the potential influence of preclinical cognitive decline, but they did not perform meta-analysis and they included retrospective studies, with an acknowledged risk of selection bias.

### 4.3. Potential Mechanisms that Might Underlie the Link between Anxiety and All-Cause Dementia

Whether anxiety is a prodromal symptom or a risk factor of dementia remains controversial. Sustained and impactful symptoms of emotional dysregulation, such as depression, anxiety or irritability, have been included in the construct of Mild Behavioral Impairment (MBI), considered a precursor of cognitive decline and dementia in adults older than 50 [38]. These symptoms could be due to neurobiological changes in particularly vulnerable regions that, in the case of Alzheimer’s Disease (AD), may precede the onset of cognitive symptoms by at least 10–20 years [39]. For example, depressive symptoms have been found to emerge approximately a decade prior to dementia diagnosis [40]. Anxiety might be also a risk factor for dementia, as indicated by one study included in our meta-analysis that found a significant increased risk of dementia for participants with anxiety after 28 years of follow-up [33]. A similar association between anxiety and dementia was also reported in the systematic review of Gimson et al. [12], where the mean interval between anxiety assessment and dementia diagnosis was more than 10 years. Thus, these findings might suggest that anxiety is acting as a very early risk factor for dementia, which could be explained by several hypothetical mechanisms: an increased risk of cardio- and cerebro-vascular events [41,42], which are themselves risk factors for vascular dementia [43] and AD [44]; raised levels of glucocorticoids [45], which increase the risk of cardiovascular diseases [46] and may promote hippocampal atrophy [47]; a decrease in brain reserve [48,49]; and an increased gut permeability [50] and changes in gut-microbiota composition [51], which have been implicated in the modulation of cognitive function and related with fragility and dementia [52]. Finally, anxiety is associated with avoidant behavior, which in turn may result in social isolation and lower levels of physical activity [53], both risk factors for dementia [54,55].

### 4.4. Strengths and Limitations

Our study has several strengths. As a MA of all available up-to-date studies of anxiety and the risk of all-cause dementia, it has greater power than individual studies and previous meta-analyses with fewer studies. Moreover, we included cohort studies mostly comprising cognitively intact community samples or studies that controlled for baseline cognition in their analyses, thus minimizing the risk of recall and selection bias. The long periods of follow-up in the studies allow a sufficient number of incident dementia cases to be observed. All the studies included in the MA reported adjusted risk estimations, thus helping to provide more accurate results. Finally, all included studies except one [31] controlled for depression at baseline. This is relevant because depression is highly associated with anxiety in elderly community samples [56], and it is a well-known risk factor for dementia [57].

Some limitations should be considered when interpreting our results. In our MA, studies assessed anxiety with different tools, and only two used clinical criteria to determine clinically relevant anxiety [13,18]. Most studies assessed anxiety with symptomatic scales, which means we cannot differentiate the presence of anxiety symptoms from anxiety disorders that may differ in psychopathology, management and course. Additionally, two studies assessed anxiety as a personality trait and not as a symptomatic state [16,32], which might explain why they did not find significant results. While heterogeneity between the studies was moderate, our sensitivity analysis showed that none of the studies had a disproportionate impact on the findings. Our results might be also influenced by the heterogeneous characteristics of the studies, although univariate meta-regressions showed that there were no significant associations between the outcome and several variables (i.e., age, gender, sample size, follow-up period or methodological quality). Despite the controversial effect of psychotropic drugs on dementia risk, [58,59,60,61], we did not take into account how taking psychotropic drugs could affect our results, because only one study assessed this variable [34]. Due to the fact that the included studies assessed risk of dementia across their entire follow-up periods, we are unable to pinpoint a time before dementia diagnosis when the association with anxiety may begin. Finally, and despite some evidence of publication bias, we found that the pooled relative risk, after correction for publication bias, was similar to the non-corrected value.

### 4.5. Clinical and Public Health Implications

Our results have several clinical implications. Anxiety is a common yet treatable mental disorder [62], despite it being sometimes difficult to identify in older adults [63]. If anxiety is indeed a risk factor for dementia, rather than a prodromal symptom, treating the condition could help prevent dementia. Benzodiazepines are commonly used to treat anxiety symptoms, but some studies have found that the use of benzodiazepines is associated with an increased risk of dementia [58,59]. However, these results are controversial [60,61] and future studies are warranted to further investigate the role of benzodiazepine intake and risk of dementia.

Epidemiological research has an important role in the study of potential modifiable risk factors of dementia, given the current absence of treatment for this disease. Our finding of a 24% increased risk of all-cause dementia for subjects with anxiety is comparable to that reported for low education as a risk factor of dementia (HR = 1.28) [3]. Considering the high proportion of older adults suffering from anxiety, our findings further indicate that nearly 4% of dementia cases would be avoided if anxiety could be prevented or treated (assuming a causal effect and unbiased estimates).

## 5. Conclusions

In conclusion, this meta-analysis adds to previous evidence on anxiety as a significant risk factor of all-cause dementia. Considering that anxiety is a common yet treatable condition among older adults, its treatment and prevention might help reduce the incidence and prevalence of dementia, as well as its personal, familiar and social burden. However, more research is needed to finally clarify whether anxiety is a cause of dementia or rather an early sign of the disease itself.

## Figures and Tables

**Table 1:** Characteristics of the studies included in the meta-analysis.

Authors, Year	Country	*N*	Follow-up, y.	Age, mean y. (SD)	Females, *n* (%)	Anxiety Measure	Dementia Criteria	Dementia Cases (*n*)	Risk Estimates (95% CI)	Statistical Model	Covariates	Quality Score
Acosta et al., 2018 [35]	Mexico	1355	3	73.6 (6.4)	1144 (62.7)	NPI-Q	DSM-IV	129	RR: 1.7 (1.2–2.5)	Poisson regression	Age, sex, education, MCI, delusions, hallucinations, depression, and aberrant motor behaviour	7
de Bruijn et al., 2014 (sample I) [13]	Netherlands	2708	17	68.6 (8.5)	1495 (55.2)	HADS	DSM-III-R	358	HR: 0.99 (0.69–1.41)	Cox regression	Age, sex, educational level (low), ApoE-ε4 and depressive symptoms.	9
de Bruijn et al., 2014 (sample II) [13]	Netherlands	3079	9	75.5 (6.2)	1810 (59.1)	DSM-IV	DSM-III-R	248	HR: 0.81 (0.50–1.30)	Cox regression	Age, sex, educational level (low), ApoE-ε4 and depressive disorder.	8
Gallacher et al., 2009 [32]	United Kingdom	755	17	NR (NR)	0 (0)	STAI-trait scale	DSM-IV	NR	OR: 1.77 (0.31–10.2)	Logistic regression	Age, Vascular risk factors, GHQ and NART	6
Kassem et al., 2017 [34]	United States	1425	5	82.8 (3.1)	1425 (100)	GAS	DSM-IV	233	OR: 1.56 (1.07–2.26)	Logistic regression	Age, education, marital status, health behaviours, medical history, psychotropic medications, depression, poor sleep.	6
Mortamais et al. 2018 [16]	France	5234	10	73.4 (5.2)	3069 (58.5)	STAI-trait scale	DSM-IV	378	HR: 1.04 (0.81–1.32)	Cox regression	Age, sex, center, smoking habits, alcohol intake, education, living alone, body mass index, history of vascular pathology, hypertension, diabetes, dyslipidemia, incapacity, MMSE at baseline and depressive symptoms.	7
Petkus et al., 2015 [33]	Sweden	1082	28	60.8 (11.1)	612 (56.6)	STAI-state scale	DSM-III, IV	172	HR: 1.48 (1.01–2.18)	Cox mixed Effects regression	Age, sex, education, physical illness, depression (average and symptoms), neuroticism	8
Santabárbara et al., 2018 [18]	Spain	4057	4.5	72.1 (9.1)	2229 (54.9)	GMS-AGECAT	DSM-IV	138	SHR: 2.74 (1.18–6.35)	Fine and Gray Regression	Age (as timescale), sex, educational level, marital status, living alone, vascular disease, hypertension, diabetes, health status, depression and cognitive status.	7
Sutin et al., 2018 [17]	United States	9913	8	67.03 (9.16)	5948 (60)	Beck Anxiety Inventory	TICSm	397	HR: 1.16 (1.04–1.28)	Cox regression	Age, sex, race, ethnicity, education, depressive symptoms, history of a mental disorder, obesity, diabetes, hypertension, smoking and physical activity	6

Abbreviations in the table: ApoE: Apolipoprotein E; DSM-III: Diagnostic and Statistical Manual, Third Edition; DSM- IV: Diagnostic and Statistical Manual, Fourth Edition; GAS: Geriatric Anxiety Scale; GHQ: General health questionnaire; GMS-AGECAT: Geriatric Mental State- Automated Geriatric Examination for Computer Assisted Taxonomy; HADS: Hospital Anxiety and Depression Scale; HR: Hazard Ratio; MCI: Mild Cognitive Impairment; NART: National adult reading test; NPI-Q: Neuropsychiatric Inventory Questionnaire; NR: not reported; OR: Odds Ratio; RR: Relative Risk; SD: Standard deviation; SHR: Subdistribution hazard ratio; STAI: State-Trait Anxiety Inventory; TICSm: modified Telephone Interview for Cognitive Status; y.:years.

**Table 2:** Univariate meta-regression results for the log(RR).

	*b*	95% CI	*p* value
Age (75 + years) *	−0.09	(−0.77; 0.59)	0.749
Female (%)	0.004	(−0.011; 0.020)	0.519
Sample size (per 1000 persons)	−0.03	(−0.10; 0.04)	0.398
Follow-up (years)	−0.006	(−0.039; 0.027)	0.681
Methodological quality (score)	−0.09	(−0.32; 0.14)	0.382

*b* = regression coefficient; 95% CI = 95% Confidence interval. * Gallacher et al. [32] not included.

## Supplementary Materials

The following are available online at https://www.mdpi.com/2077-0383/9/6/1791/s1, Table S1: Search strategy, Table S2: Quality assessment of studies in the meta-analysis using the Newcastle-Ottawa scale (NOS), Figure S1: Sensitivity analysis, Figure S2: Trim & Fill plot.

## References

[1] World Health Organization (2015). First WHO Ministerial Conference on Global Action against Dementia.

[2] Birdi R., Stephan B.C.M., Robinson L., Davis D. (2015). Can we influence the epidemiology of dementia? Prospectives from population based studies. Postgrad. Med. J..

[3] Ritchie K., Ritchie C., Berr C., Artero S., Ancelin M.L. (2010). Designing prevention programmes to reduce incidence of dementia: Prospective cohort study of modifiable risk factors. BMJ.

[4] Deckers K., van Boxtel M.P., Schiepers O.J., de Vugt M., Muñoz-Sánchez J.L., Anstey K.J., Brayne C., Dartigues J.F., Engendal K., Kivipelto M. (2015). Target risk factors for dementia prevention: A systematic review and Delphi consensus study on the evidence from observational studies. Int. J. Geriatr. Psychiatry.

[5] Patterson C. World Alzheimer Report 2018. The State of the Art of Dementia Research: New Frontiers. https://www.alz.co.uk/research/WorldAlzheimerReport2018.pdf.

[6] Matthews F.E., Arthur A., Barnes L.E., Bond J., Jagger C., Robinson L., Brayne C., Medical Research Council Cognitive Function and Ageing Collaboration (2013). A two-decade comparison of prevalence of dementia in individuals aged 65 years and older from three geographical areas of England: Results of the Cognitive Function and Ageing Study I and II. Lancet.

[7] Lobo A., Saz P., Marcos G., Dia J.L., De-la-Cámara C., Ventura T., Montañes J.A., Lobo-Escolar A., Aznar S., ZARADEMP Workgroup (2007). Prevalence of dementia in a southern European population in two different time periods: The ZARADEMP Project. Acta Psychiatr. Scand..

[8] Schrijvers E.M.C., Verhaaren B.F., Koudstaal P.J., Hofman A., Ikram M.A., Breteler M.M. (2012). Is dementia incidence declining? Trends in dementia incidence since 1990 in the Rotterdam Study. Neurology.

[9] Qiu C., von Strauss E., Backman L., Winblad B., Fratiglioni L. (2013). Twenty-year changes in dementia occurrence suggest decreasing incidence in central Stockholm, Sweden. Neurology.

[10] Livingston G., Sommerlad A., Orgeta V., Costafreda S.G., Huntley J., Ames D., Ballard C., Banerjee S., Burns A., Cohen-Mansfield J. (2017). Dementia prevention, intervention, and care. Lancet.

[11] Prince M., Albanese E., Guerchet M., Prina M. (2014). World Alzheimer Report 2014. Dementia and Risk Reduction. An Analysis of Protective and Modifiable Risk Factors. Alzheimer’s Disease International. http://www.alz.co.uk/research/WorldAlzheimerReport2014.pdf.

[12] Gimson A., Schlosser M., Huntley J.D., Marchant N.L. (2018). Support for midlife anxiety diagnosis as an independent risk factor for dementia: A systematic review. BMJ Open.

[13] De Bruijn R.F., Direk N., Mirza S.S., Hofman A., Koudstaal P.J., Tiemeier H., Ikram M.A. (2014). Anxiety is not associated with the risk of dementia or cognitive decline: The Rotterdam Study. Am. J. Geriatr. Psychiatry.

[14] Gulpers B., Ramakers I., Hamel R., Köhler S., Oude Voshaar R., Verhey F. (2016). Anxiety as a Predictor for Cognitive Decline and Dementia: A Systematic Review and Meta-Analysis. Am. J. Geriatr. Psychiatry.

[15] Santabárbara J., Lipnicki D.M., Villagrasa B., Lobo E., Lopez-Anton R. (2019). Anxiety and risk of dementia: Systematic review and meta-analysis of prospective cohort studies. Maturitas.

[16] Mortamais M., Abdennour M., Bergua V., Tzourio C., Berr C., Gabelle A., Akbaraly T.N. (2018). Anxiety and 10-Year Risk of Incident Dementia-An Association Shaped by Depressive Symptoms: Results of the Prospective Three-City Study. Front. Neurosci..

[17] Sutin A.R., Stephan Y., Terracciano A. (2018). Psychological Distress, Self-Beliefs, and Risk of Cognitive Impairment and Dementia. J. Alzheimers Dis..

[18] Santabárbara J., Lopez-Anton R., De la Cámara C., Lobo E., Gracia-García P., Villagrasa B., Bueno-Notivol J., Marcos G., Lobo A. (2019). Clinically significant anxiety as a risk factor for dementia in the elderly community. Acta Psychiatr. Scand..

[19] Moher D., Liberati A., Tetzlaff J., Altman D.G., The PRISMA Group (2009). Preferred Reporting Items for Systematic Reviews and Meta-Analyses: The PRISMA Statement. PLoS Med..

[20] Wells G.A., Shea B., O’Connell D., Peterson J., Welch V., Losos M. (2016). The Newcastle-Ottawa Scale (NOS) for Assessing the Quality of Nonrandomised Studies in Meta-Analyses. http://www.ohri.ca/programs/clinical_epidemiology/oxford.asp.

[21] Sánchez-Meca J., Marín-Martínez F., Chacón-Moscoso S. (2003). Effect-size indices for dichotomized outcomes in meta-analysis. Psychol. Methods.

[22] Cohen J. (1977). Statistical Power Analysis for the Behavioural Sciencies.

[23] Higgins J.P., Thompson S.G., Deeks J.J., Altman D.G. (2003). Measuring inconsistency in meta-analyses. BMJ.

[24] Thompson S.G., Higgins J.P. (2002). How should meta-regression analyses be undertaken and interpreted?. Stat. Med..

[25] Rosenberg M.S. (2005). The file-drawer problem revisited: A general weighted method for calculating fail-safe numbers in meta-analysis. Evolution.

[26] Higgins J.P.T., Green S. (2011). Cochrane Handbook for Systematic Reviews of Interventions.

[27] Sterne J.A., Sutton A.J., Ioannidis J.P., Terrin N., Jones D.R., Lau J., Carpenter J., Rücker G., Harbord R.M., Schmid C.H. (2011). Recommendations for examining and interpreting funnel plot asymmetry in metaanalyses of randomised controlled trials. BMJ.

[28] Duval S., Tweedie R. (2000). Trim and fill: A simple funnel-plot-based method of testing and adjusting for publication bias in meta-analysis. Biometrics.

[29] Miettinen O.S. (1974). Proportion of disease caused or prevented by a given exposure, trait or intervention. Am. J. Epidemiol..

[30] Daly L.E. (1998). Confidence limits made easy: Interval estimation using a substitution method. Am. J. Epidemiol..

[31] Villagrasa B., Olaya B., López-Antón R., de la Cámara C., Lobo A., Santabárbara J. (2018). Prevalence of anxiety disorder among older adults in spain: A meta-analysis. J. Affect. Disord..

[32] Gallacher J., Bayer A., Fish M., Pickering J., Pedro S., Dunstan F., Ebrahim S., Ben-Shlomo Y. (2009). Doesanxietyaffectrisk of dementia? Findingsfromthe Caerphilly Prospective Study. Psychosom. Med..

[33] Petkus A.J., Reynolds C.A., Wetherell J.L., Kremen W.S., Pedersen N.L., Gatz M. (2016). Anxiety is associated with increased risk of dementia in older Swedish twins. Alzheimers Dement..

[34] Kassem A.M., Ganguli M., Yaffe K., Hanlon J.T., Lopez O.L., Wilson J.W., Ensrud K., Cauley J.A., Study of Osteoportic Fractures (SOF) Research Group (2018). Anxiety symptoms and risk of dementia and mild cognitive impairment in the oldest old women. Aging Ment. Health.

[35] Acosta I., Borges G., Aguirre-Hernández R., Sosa A.L., Prince M., 10/66 Dementia Research Group (2018). Neuropsychiatric symptoms as risk factors of dementia in a Mexican population: A 10/66 Dementia Research Group study. Alzheimers Dement..

[36] American Psychiatric Association (2000). Diagnostic and Statistical Manual of Mental Disorders: DSM-IV-TR.

[37] Marcos G., Santabárbara J., López-Antón R., de la Cámara C., Gracia-García P., Lobo E., Pirez G., Menchón J.M., Palomo T., Stephan B.C.M. (2016). Conversion to dementia in mild cognitive diagnosed with DSM-5 criteria and with Petersen’s criteria. Acta Psychiatr. Scand..

[38] Ismail Z., Gatchel J., Bateman D.R., Barcelos-Ferreira R., Cantillon M., Jaeger J., Donovan N.J., Mortby M.E. (2018). Affective and emotional dysregulation as pre-dementia risk markers: Exploring the mild behavioral impairtment sysmptoms of depression, anxiety, irritability and euphoria. Int. Psychogeriatr..

[39] Long J.M., Holtzman D.M. (2019). Alzheimer Disease: An update on pathobiology and treatment strategies. Cell.

[40] Singh-Manoux A., Dugravot A., Fournier A., Abell J., Ebmeier K., Kivimäki M., Sabia S. (2017). Trajectories of depressive symptoms before diagnosis of dementia: A 28-year follow-up study. JAMA Psychiatry.

[41] Emdin C.A., Oduyato A., Wong C.X., Tran J., Hsiao A.J., Hunn B.H. (2016). Meta-analysis of anxiety as a risk for cardiovascular disease. Am. J. Cardiol..

[42] Batelaan N.M., Seldernrijk A., Bot M., van Balkom A.J., Pennix B.W. (2016). Anxiety and new onset of cardiovascular disease: Critical review and meta-analysis. Br. J. Psychiatry.

[43] O’Brien J.T., Thomas A. (2015). Vascular dementia. Lancet.

[44] Gottesman R.F., Schneider A.L., Zhou Y., Coresh J., Green E., Gupta N., Knopman D.S., Mintz A., Rahmim A., Sharrett A.R. (2017). Association between midlife vascular risk factors and estimated brain amyloid deposition. JAMA.

[45] Raglan G.B., Schmidt L.A., Schulkin J. (2017). The role of glucocorticoids and corticotropin-releasing hormone regulation on anxiety symptoms and response to treatment. Endocr. Connect..

[46] Burford N.G., Webster N.A., Cruz-Topete D. (2017). Hypothalamic-Pituitary-Adrenal axis modulation of glucocorticoids in the cardiovascular system. Int. J. Mol. Sci..

[47] Sapolsky R.M. (2000). Glucocorticoids and hippocampal atrophy in neuropsychiatric disorders. Arch. Gen. Psychiatry.

[48] Perna G., Iannone G., Alciati A., Caldirola D. (2016). Are anxiety disorders associated with accelerated aging? A focus on neuroprogression. Neural Plast..

[49] Vance D.E., Roberson A.J., McGuiness T.M., Fazeli P.L. (2010). How neuroplasticity and cognitive reserve protect cognitive functioning. J. Psychosoc. Nurs. Ment. Health Serv..

[50] Stevens B.R., Goel R., Seungbum K., Richards E.M., Holtbert R.C., Pepine C.J., Raizada M.K. (2018). Increased human intestinal barrier permeability plasma biomarkers zonulin and FABP2 correlated with plasma LPS and altered gut microbiome in anxiety or depression. Gut.

[51] De Palma G., Lynch M.D.J., Lu J., Dang V.T., Deng Y., Jury J., Umeh G., Miranda P.M., Pigrau Pastor M., Sidani S. (2017). Transplantation of fecal microbiota from patients with irritable bowel syndrome alters gut function and behavior in recipient mice. Sci. Transl. Med..

[52] Ticinesi A., Tana C., Nouvenne A., Prati B., Lauretani F., Meschi T. (2018). Gut microbiota, cognitive frailty and dementia in older individuals: A systematic review. Clin. Interv. Aging.

[53] Celano C.M., Daunis D.J., Lokko H.N., Campbell K.A., Huffman J.C. (2016). Anxiety disorders and cardiovascular disease. Curr. Psychiatry Resp..

[54] Kuiper J.S., Zuidersma M., Oude Voshaar R.C., Zuidema S.U., van den Heuvel E.R., Stolk R.P., Smidt N. (2015). Social relationships and risk of dementia: A systematic review and meta-analysis of longitudinal cohort studies. Ageing Res. Rev..

[55] Laurin D., Verreault R., Lindsay J., MacPherson K., Rockwood K. (2001). Physical activity and risk of cognitive impairtment and dementia in elderly persons. Arch. Neurol..

[56] Braam A.W., Copeland J.R., Delespaul P.A., Beekman A.T., Como A., Dewey M., Fichter M., Holwerda T.J., Lawlor B.A., Lobo A. (2014). Depression, subthreshold depression and comorbid anxiety symptoms in older Europeans: Results from the EURODEP concerted action. J. Affect. Disord..

[57] Cherbuin N., Kim S., Anstey K.J. (2015). Dementia risk estimates associated with measures of depression: A systematic review and meta-analysis. BMJ Open.

[58] He Q., Chen X., Wu T., Li L., Fei X. (2019). Risk of dementia in long-term benzodiazepine users: Evidence from a meta-analysis of observational studies. J. Clin. Neurol..

[59] Lucchetta R.C., da Mata B.P.M., Mastroianni P.C. (2018). Association between development of dementia and use of benzodiazepines: A systematic review and meta-analysis. Pharmacotherapy.

[60] Richardson K., Mattishen K., Loye Y.K., Steel N., Fox C., Grossi C.M., Bennett K., Maidment I., Boustani M., Matthews F.E. (2019). History of benzodiazepine prescriptions and risk of dementia: Possible bias due to prevalent users and covariate measurement timing in a nested case-control study. Am. J. Epidemiol..

[61] Penninkilampi R., Eslick G.D. (2018). A systematic review and meta-analysis of the risk of dementia associated with benzodiazepines use, after controlling for protopathic bias. CNS Drugs.

[62] Bandelow B., Michaelis S. (2015). Epidemiology of anxiety disorders in the 21st century. Dialogues Clin. Neurosci..

[63] Therrien Z., Hunsley J. (2012). Assessment of anxiety in older adults: A systematic review of commonly used measures. Aging Ment. Health.

