# Supplementary material for: Does Anxiety Increase the Risk of all-Cause Dementia? An Updated Meta-Analysis of Prospective Cohort Studies
Source: J Clin Med. 2020 Jun 9;9(6):1791. doi: 10.3390/jcm9061791 (PMC7355582; doi:10.3390/jcm9061791)
Supplement: Supplementary file 1 [file jcm-09-01791-s001.pdf]

Supplementary table S1. Search strategy.

Pubmed:

("anxiety disorders"[mesh] OR "anxiety"[mesh] OR "anxiety"[tiab] OR “neuropsychiatric symptoms” [tiab]) AND (“dementia”[mesh] OR “dementia”[tiab] OR dement*) AND ("cohort studies"[Mesh] OR "epidemiologic stud*"[tiab] OR cohort*[tiab] OR “longitudinal stud*”[tiab] OR prospective[tiab] OR “risk”[tiab] OR “incidence”[Mesh]) AND ("2018/01/19"[PDAT] : "2020/01/06"[PDAT]) AND English[lang]

WoS:

(AB=("anxiety disorder*" OR "anxiety" OR “neuropsychiatric symptoms”) OR TI=("anxiety disorder*" OR "anxiety" OR “neuropsychiatric symptoms”) OR TS=("anxiety disorder*" OR "anxiety" OR “neuropsychiatric symptoms”)) **AND** (AB=(“dementia” OR dement*) OR TI=(“dementia” OR dement*) OR TS=(“dementia” OR dement*)) **AND** (AB=(“cohort” OR "incidence" OR "epidemiology" OR “risk” OR “case-control” OR case control) *OR* TI=(“cohort” OR "incidence" OR "epidemiology" OR “risk” OR “case-control” OR case control) *OR* TS=(“cohort” OR "incidence" OR "epidemiology" OR “risk” OR “case-control” OR case control))

Supplementary Table S2. Quality assessment of studies in the meta-analysis using the Newcastle-Ottawa scale (NOS).

	Selection				Comparability		Outcome			Overall Quality Score (Maximum = 9)
	**1**	**2**	**3**	**4**	**5A**	**5B**	**6**	**7**	**8**	
**Acosta et al., 2018** [35]	*	*	*	*	*	*	*	-	-	7
**de Bruijn et al., 2014 (sample I)** [13]	*	*	*	*	*	*	*	*	*	9
**de Bruijn et al., 2014 (sample II)** [13]	*	*	*	*	*	*	*	-	*	8
**Gallacher et al., 2009** [32]	-	*	*	-	-	*	*	*	*	6
**Kassem et al., 2017** [34]	-	*	*	*	-	*	*	-	*	6
**Mortamais et al., 2018** [16]	*	*	*	*	*	*	-	*	-	7
**Petkus et al., 2015** [33]	*	*	*	*	*	*	*	*	-	8
**Santabárbara et al., 2018** [18]	*	*	*	*	*	*	-	-	*	7
**Sutin et al., 2018** [17]	*	*	-	-	*	*	*	-	*	6

NOS items: 1. Truly representative of the exposed cohort. 2. Non-exposed participants from same community as exposed participants 3. Ascertainment of exposure (Secured records or structured interview) 4. Demonstration that outcome of interest was not present at start of study (only incident cases of dementia). 5. Comparability of cohorts on the basis of the design or analysis (5A. Study controls for age and sex 5B. Study controls for any additional factor: education attainment, depression, physical inactivity, diabetes, obesity, smoking or hypertension) 6. Quality of outcome assessment (Independent blind assessment or record linkage) 7. Follow-up long enough for dementia to occur (≥ 10 years) 8. Complete follow-up (all participants are accounted for or subjects lost to follow-up unlikely to introduce bias).

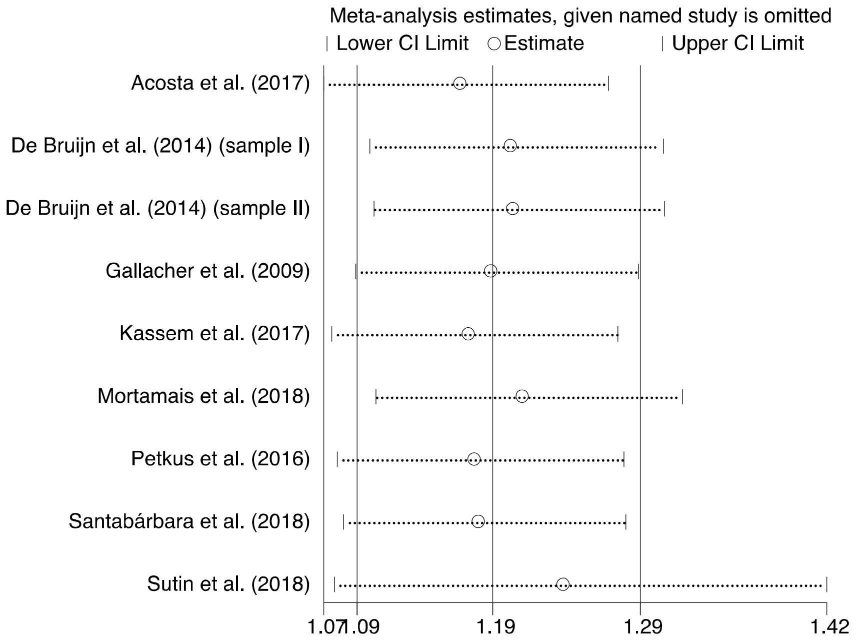

Supplementary Figure S1. Sensitivity plot.

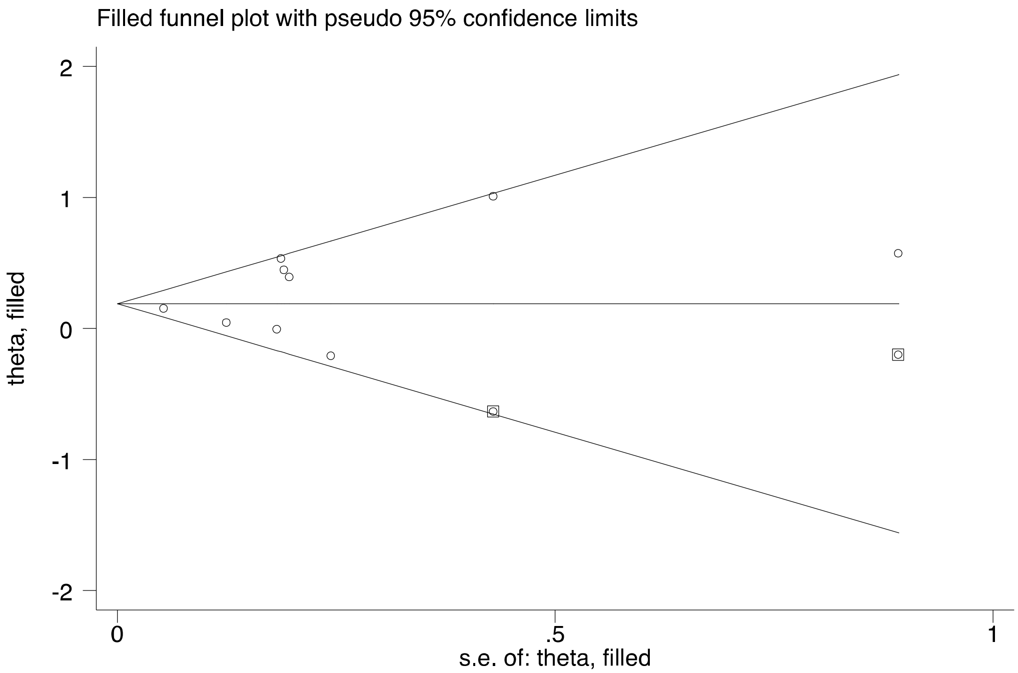

Supplementary Figure S2. Trim & Fill plot.
